# Approaches for enhancing patient-reported experience measurement with ethnically diverse communities: a rapid evidence synthesis

**DOI:** 10.1186/s12939-024-02107-5

**Published:** 2024-02-12

**Authors:** Reema Harrison, Maha Pervaz Iqbal, Upma Chitkara, Corey Adams, Ashfaq Chauhan, Rebecca Mitchell, Elizabeth Manias, Megan Alston, Anne Marie Hadley

**Affiliations:** 1 Centre for Health Systems and Safety Research, Australian Institute of Health Innovation, Faculty of Medicine, Health and Human Sciences- Macquarie University, Level 6, 75 Talavera Road, Sydney, NSW 2109 Australia; 2 Centre for Healthcare Resilience and Implementation Science, Australian Institute of Health Innovation, Faculty of Medicine, Health and Human Sciences Macquarie University, Level 6, 75 Talavera Road, Sydney, NSW 2109 Australia; 3 School of Nursing and Midwifery, Monash University, Melbourne, VIC 3800 Australia; 4 Elevating the Human Experience Program, NSW Ministry of Health, Sydney, NSW 2065 Australia

**Keywords:** Patient reported experience measures, Multicultural health, Patient experience, Patient satisfaction, Rapid evidence appraisal, Diversity, Ethnicity

## Abstract

**Background:**

Patient-reported experience measures (PREMs) are used to drive and evaluate unit and organisational-level healthcare improvement, but also at a population level, these measures can be key indicators of healthcare quality. Current evidence indicates that ethnically diverse communities frequently experience poorer care quality and outcomes, with PREMs data required from this population to direct service improvement efforts. This review synthesises evidence of the methods and approaches used to promote participation in PREMs among ethnically diverse populations.

**Methods:**

A rapid evidence appraisal (REA) methodology was utilised to identify the disparate literature on this topic. A search strategy was developed and applied to three major electronic databases in July 2022 (Medline; PsycINFO and CINAHL), in addition to websites of health agencies in Organisation for Economic Co-operation and Development countries via grey literature searches. A narrative evidence synthesis was undertaken to address the review question.

**Results:**

The review resulted in 97 included studies, comprised 86 articles from electronic database searches and 11 articles from the grey literature. Data extraction and synthesis identified five strategies used in PREM instruments and processes to enhance participation among ethnically diverse communities. Strategies applied sought to better inform communities about PREMs, to create accessible PREMs instruments, to support PREMs completion and to include culturally relevant topics. Several methods were used, predominantly drawing upon bicultural workers, translation, and community outreach to access and support communities at one or more stages of design or administration of PREMs. Limited evidence was available of the effectiveness of the identified methods and strategies. PREMs topics of trust, cultural responsiveness, care navigation and coordination were identified as pertinent to and frequently explored with this population.

**Conclusions:**

The findings provide a basis for a maturity model that may guide change to increase participation of ethnically diverse communities in PREMs. In the short-medium term, health systems and services must be able to recognise and respond to cultural and linguistic diversity in the population when applying existing PREMs. In the longer-term, by working in collaboration with ethnically diverse communities, systems and services may co-create adapted or novel PREMs that tackle the factors that currently inhibit uptake and completion among ethnically diverse communities.

## Background

Patient Reported Experience Measures (PREMs) are now among the key indicators of performance used to determine healthcare value [1]. PREMs produce local, service and system-level performance data that are essential to direct quality improvement and service development [1]. Inclusive PREMs that capture data from communities who have high healthcare utilisation and poor healthcare outcomes are therefore important to determine their experiences, and target for improvements [2]. Continued underrepresentation of people from ethnically diverse communities in PREMs data means that quality of care concerns from these communities are not identified and addressed.

People from ethnically diverse backgrounds, who speak a language other than a national language at home, have one or both parents born overseas, and/or have low proficiency in the national language/s, experience higher rates of healthcare-associated harm and preventable hospitalisations than the general population [3]. Understanding the experiences of people from ethnically diverse communities via PREMs provides an avenue to drive person-centric improvements to redress this inequity in service delivery and care outcomes. Yet limited accessibility of PREMs in terms of their structure, content and approaches to administration can prohibit their completion to improve care for people from ethnically diverse backgrounds, along with several other priority populations.

People from ethnically diverse backgrounds face specific barriers in accessing and completing PREMs that are subject to intra- and inter-group variation. Factors such as language proficiency, digital and health literacy [4], trust in government, culturally inappropriate content and limited resources to support participation create barriers for people from ethinically diverse backgrounds to participate in PREMs [5]. Widely used PREMs instruments are closed-item surveys, include technical and complex language and phrasing, and contain between 50–80 items [6, 7].

Targeted strategies and methods to increase uptake and completion of PREMs among ethnically diverse communities may contribute to reducing barriers to participation [8]. Synthesising evidence from existing studies that have captured patient-reported experiential data provides insight into the strategies and methods that have been used and may be effective in increasing participation of ethnically diverse communities in PREMs. This knowledge may inform population-based PREMs instruments and data collection approaches. Therefore, the aim of this review was to identify evidence in the peer-reviewed and grey literature of the strategies and methods employed in patient-reported experience measurement with people from ethnically diverse backgrounds to inform policy and practice.

## Methods

A rapid evidence appraisal (REA) methodology was utilised to address the review objective because this project was undertaken to inform policy and practice for NSW Ministry of Health, Australia. REA is widely applied to answer policy-related questions that require expansive literature to be explored to answer a focused question within a limited timeframe [9]. REA rigorously follows established systematic review methodology to search and appraise existing evidence, limiting selected aspects of the review process to shorten the review timespan while still enabling the depth of current knowledge to be appraised [10]. In this REA, the search was limited to three electronic databases to enable a breadth of literature to be explored including grey material. The Centre for Evidence-Based Medicine (CEBM) guideline for REAs was followed [10].

To ensure a search strategy that was both sensitive and specific, comprehensive search strategies were developed by a medical information specialist for the electronic databases of published literature and for use with grey literature. The search strategy was applied to the following electronic databases in June 2022 by the medical information specialist: Medline; PsycINFO and CINAHL. A research team member (MPI) applied the same search terms to the websites of health agencies in Organisation for Economic Co-operation and Development (OECD) countries in which understanding and improving patient experience has been identified as a key outcome in relation to value-based care. In addition, the Preferred Reporting Items for Systematic Reviews and Meta-Analyses—PRISMA statement—was used to guide the reporting of this REA [11].

### Inclusion criteria

Articles were included if they met the following inclusion criteria:

- Types of publication: Publications available in English, reporting original primary empirical or theoretical work, and published from the year 2000 onwards, which is contemporaneous with exploration of patient experience in health settings.
- Types of settings: Any healthcare setting, including but not limited to public or private hospitals, day procedure centres, general practice or other primary/community care in OECD countries.
- Types of study design: Conceptual, theoretical, quantitative, or qualitative studies of any research design.
- Types of population: Health care consumers from ethnically diverse backgrounds who access health services were included; defined as born overseas or who have one or more parents born overseas in a country where English is not a national language, and/or who speak a language other than English at home; and/or who have low English language proficiency.
- Interventions: Strategies or methods to increase uptake and/or completion of PREMs.
- Outcomes: PREMs included any form of data “from patients on what happened to them in the course of care or treatment” were eligible for inclusion [12]. In this review, the focus was on experiences of a healthcare encounter or a service rather than general attitudes or perceptions of healthcare.

### Exclusion criteria

Articles were excluded if they reported general beliefs or attitudes about healthcare rather than experiences of a care episode, along with those that did not meet the above criteria, or reported reviews, protocols, opinion, or editorial pieces.

### Study identification and selection

Covidence systematic review software (Veritas Health Innovation, Melbourne, Australia) was used for study screening and management. Two reviewers (MPI, UC) screened the titles and abstracts in Covidence against the eligibility criteria. Full-text documents were obtained for all potentially relevant articles. The eligibility criteria were then applied to the articles by three reviewers (AC, MPI, UC). Four team members then met to finalise the eligible articles for inclusion across the published and grey literature (MPI, RH, RM, EM).

### Data extraction and synthesis

A narrative evidence synthesis was undertaken to address the project aim of collating established experience measurement approaches and the impact of these methods on participation of ethnically diverse individuals [13]. Separate data extraction tools were developed for full-text articles and the grey literature and each tool was used to extract relevant information using a data extraction form created in MS Excel. Evidence synthesis occurred in stages and was conducted using a team-based approach involving seven members of the research team (RH, MPI, AC, CA, UC, RM and EM). Following the tabulation of initial descriptions of the included studies, their approaches and techniques, and the resulting impact on participation (where reported), team members individually reviewed the included articles. The group met to discuss key findings, to explore commonalities in current approaches and techniques that have been successfully applied, and to identify any challenges and mitigation strategies adopted. Through this group discussion, initial themes were generated and used to describe the evidence available. Two research team members developed the results content and shared this content with the wider group to further refine the identified themes.

## Results

### Search results

**Fig. 1:**
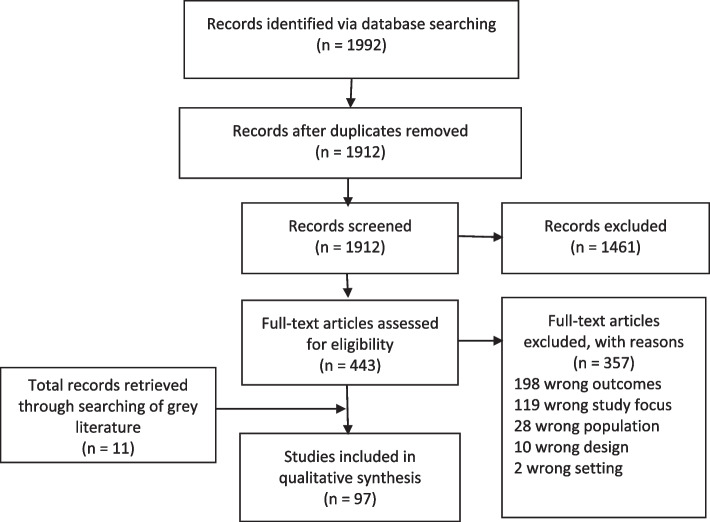
Prisma flow diagram CALD report

The systematic database search retrieved 1992 articles. After removal of 80 duplicates, 1912 articles remained. A total of 1461 articles were excluded after title and abstract screening. The remaining 443 articles underwent full-text review, of which 357 were excluded. A total of 97 documents were included, composed of 86 peer-reviewed journals and 11 documents from grey literature. Figure 1 demonstrates the search and selection process. Descriptions of eligible studies and results were tabulated. Tables 1, 2 and 3 show summaries of the included quantitative, mixed methods and qualitative articles from the electronic data search respectively. Table 4 shows the summary of grey literature articles.

**Table 1:** Summary of included studies: quantitative *N* = 27

	**Author**	**Year**	**Country**	**Setting**	**Aspect measured in the Patient Reported Experience measure: topics and relevant questions**	**Sample and population**	**Description of qualitative and quantitative data collection (i.e. survey)**	**Specific strategies employed to improve participation of CALD population**	**How were recruitment sites identified and examples of places**	**Evidence of effectiveness of strategy to increase CALD patient participation**
1.	Bockey	2020	Germany	Primary care	Integrated health care facility (ICF)	*N* = 102 patients	Cross sectional study Quantitative study: Questionnaire with open and closed ended questions Questions derived from the validated German ZUF-8 client satisfaction	• Questionnaire was translated into five key languages spoken including English, German, Arabic, French and Chinese • English and German language questionnaires were offered verbally as face-to-face interviews • peers were permitted to assist with the completion of questionnaires in other languages • The questions were pilot tested with the Integrated care facility staff members • Intra-method mixing, a technique that uses both open and closed ended items to achieve more comprehensive data	Participants (asylum seekers and refugees) living in the Integrated care facility were recruited	Response rate -60%
2.	Boutziona	2020	Greece	Hospital	Emergency department experience Specific questions: Do you visit Albania looking for medical care? Do you think it is better to address a health problem in Greece than in Albania?	Snowballing sampling- *N* = 167 adult patients of Albanian origin completed the questionnaire	Cross sectional study Quantitative: Survey	• A pilot questionnaire was initially developed and tested on a sample of 15 patients (Albanian immigrants), to determine its applicability and validity to the specific population • The questionnaire was cross translated from Greek into Albanian, and vice-versa, in order to ensure coherence between the Greek and Albanian versions • Eligible patients were asked if they would like to participate in the study while waiting for their test results	adult patients of Albanian origin who visited the ED of a tertiary general hospital was invited to participate	Response rate 83.5% (167/200 surveys completed) Although 75% of participants reported they had good knowledge of the Greek language and could use it to function in their daily lives, only 27.5% of them chose to complete the questionnaire in Greek
3.	Cook	2015	USA	Primary care	Integrated care: health centre medical home care	*N* = 488 patients surveys	Patient experience Quantitative Study: Clinician and Group Surveys Consumer Assessment of Healthcare Providers and Systems (CG-CAHPS) and the CG-CAHPS PCMH item set	• As many surveyors were multi or bilingual, patients were surveyed in their chosen language of English, Spanish or Haitian Creole • Patients were advised that they would receive a $5 Wal-Mart gift card for completing the survey • The project team developed an initial question set. The final tool was pilot tested with four patients, which resulted in minor revisions to wording	All surveys were conducted face-to-face at the Health Centres by faculty and students from a Master of Public Health program	Response rate: 96.6% (488/505 surveys completed)
4.	Cook	2016	USA	Primary Care	Integrated care: Patient-Centered Medical Home (PCMH)	*N* = 351 patients	cross-sectional study design Quantitative: 36 item questionnaire designed from previously validated questionnaires	• The questionnaire was translated into Spanish and Haitian Creole by native language speakers to improve the cultural appropriateness of survey questions • Administered questionnaire online and face to face in different languages • Patients, if they asked, had access to a printout of the questions to follow along with the surveyor • Patients received a $5 gift card for completing the questionnaire	At each of the four sites, surveyors had full access to screen and recruit patients from waiting rooms, using a convenience sampling approach. Surveyors approached adult patients who were not otherwise engaged (eg, talking on cell phone; sleeping)	Estimated 90%
5.	Detollenaere	2018	Europe	Primary care	Patient satisfaction with general practice	Patients completed the questionnaire Europe wide	Cross-sectional study Quantitative questionnaire	• Social groups were identified according to four patient characteristics: education, household income, ethnicity and gender (male/female)	Patients sitting in the waiting room of the GP were asked to participate	Response rate was 74.1%
6.	Eskes	2013	USA	Primary care	Spanish Patient satisfaction with primary care	Consecutive sampling	Quantitative questionnaire	• Spanish version of the survey used • Survey shortened in order to be completed in clinical setting • Participants were provided with a cover letter in Spanish explaining that their participation in the survey was voluntary	Patients recruited from community care clinics	Not reported
7.	Gurbuz	2019	Germany	Hospital	Patient satisfaction: Maternity care	*N* = 410 patients	Quantitative questionnaire. A modified version of the Migrant Friendly Maternity Care Questionnaire (MFMCQ)	• Questionnaire translated in in German, English, French, Spanish, Arabic and Turkish was used • Offering to complete the questionnaire in an interview	Patient invited from sites where they had given birth	The overall response rate of evaluable questionnaires was 58.4% (410 out of 701 women)
8.	Henderson	2018	UK	Hospital	Patient satisfaction with Maternity Care	Random sample *N* = 5332 patients	Cross sectional study Quantitative questionnaire	• Patients were mailed the questionnaire • Invitation to participate included a sentence in 18 different languages which encouraged them to call a Freephone number to enable them to complete the questionnaire by interview or through an interpreter if preferred	none	5332 women responded to the survey (a usable response rate of 54%)
9.	LaGrandeur	2018	USA	Community setting	Patient experience with student directed free clinic for pediatric patients	*N* = 63 patients	Quantitative questionnaire Parents of patients were surveyed using an instrument created by Commitment to Underserved People (CUP) students through small group discussion in 2017 for use in the TotShots program	• Offered in English and Spanish	Patients recruited using social media, and email communication with school district social workers, coaches, and nurses	Response rate 95.4%
10.	Lim	2019	Australia	Hospital	Care coordination and health literacy	*N* = 68 patients and *n* = 8 carers	Cross sectional study Quantitative questionnaire Health Literacy and Cancer Care Coordination questionnaires	• Chinese versions of both HLQ and CCCQ, which were translated using the forward‐backward procedure were used • Questionnaire pilot tested with leaders of Chinese community cancer support organisations in Sydney, Australia to ensure clarity and cultural appropriateness	Participants recruited if they attended Chinese community cancer support organisations or cancer treatment centres across the wider Sydney region	None reported
11.	Lindberg	2019	Denmark	Hospital	Mental health treatment	*n* = 686	Cross sectional study Quantitative questionnaire- patient satisfaction questionnaire	• Questionnaire developed after clinical experience with a multicultural patient population • The questionnaire was forward–backward translated from Danish to five additional languages: Arabic, Bosnian, English, Persian and Russian	Participants received the questionnaire after the last treatment session and could complete it immediately or at home and return it by mail. If participants missed their last session, the questionnaire and a stamped return envelope were mailed to them	Response rate 76.6%
12.	Mander	2016	Australia	Hospital	Maternity care	*N* = 655 women with CALD background	Cross sectional study Quantitative questionnaire- Having a Baby in Queensland Survey 2012	• Multiple formats of the survey: The survey could be completed on paper (Returned via mail with provided reply-paid envelope) or online • Survey could also be completed over the telephone with a trained female interviewer and translator if required • Multiple language instructions: Instructions for survey participation and completion were provided in English and 19 other languages • Special questions to identify CALD population: (“Where were you born?” “Do you identify with any cultural group(s) or ethnicity?”; “What language(s) do you speak at home?”)	None	None
13.	Martino	2022	USA	Community setting	Integrated care: Healthcare experience	Hispanic patients living in rural residence experience with healthcare	Quantitative questionnaire- Healthcare Providers and Systems (CAHPS) survey	• Multiple languages and multiple formats: The surveys were administered in English and Spanish by mail, with bilingual telephone follow-up of nonrespondents • Specific questions to identify race /ethnicity: Are you of Hispanic or Latino origin or descent?	none	Response rate: 42–43%
14.	Moroz	2003	USA	Community setting	Healthcare experience: convalescence care	*N* = 70 patients	Quantitative questionnaires	• The survey available in Chinese language • Patients could be assisted in completing the survey	Via patient brochures Outreach specialist visited 12 community sites Relevant doctors were provided with information about the program	none
15.	Nayfeh	2021	Canada	Hospital	End of life care for patients with diverse backgrounds	*N* = 1543	Quantitative questionnaires: End-of-Life Satisfaction Survey was used to measure satisfaction with the quality of inpatient end of-life care from the perspective of next-of-kin of recently deceased patients at Sunnybrook Health Sciences Centre in Toronto, Ontario	• The items included: patient race/ethnicity (Caucasian, Caucasian, Mediterranean, Black, East Asian, South Asian, Southeast Asian, Middle Eastern, Hispanic, First Nations, and other); patient religion [Atheist, Buddhist, Christian (all denominations), Hindu, Jehovah’s Witness, Jewish, Mormon, Muslim, Sikh, no religion, other]; level of religiosity/spirituality; and preferred spoken language • Invitation letter accompanying the survey explained the confidential and voluntary nature of the request. One reminder survey was sent three weeks after the initial mail-out to those who did not respond	none	Response rate was 37.7%
16.	Olausson	2016	Sweden	Public dental services	Dental services		Quantitative questionnaires: Dental Visit Satisfaction Scale (DVSS)	• Multiple languages: The questionnaires were available in English, Swedish, Arabic and Farsi • At the clinics all patients aged 18 or older were asked to participate. Most completed the questionnaires in the waiting room prior to treatment, but five people answered the questionnaires at home and mailed them back • The participants were asked about the following background factors: Gender; Age; Education; Dental habits; Country of origin; Skills in Swedish language	Two of the clinics were located in multicultural areas with a high proportion of foreign-born patients	response rate was 74%
17.	Parast	2022	USA	Hospital	Emergency department—racial/ethnic differences in the experience of care received during an ED visit	*N* = 16,006 eligible patients discharged from the 50 hospitals were randomly sampled survey modes	Quantitative questionnaires: Emergency Department Patient Experience of Care (EDPEC) DTC Survey	• Different modes of survey administration: mail only, telephone only, or mixed mode (mail with telephone follow-up); the survey was conducted in English • Linear regression used to measure the differences in patient experiences based on racial /ethnic group	None reported	response rate: 20.25%
18.	Pinder	2016	UK	Hospital	Patients experiences of receiving care for cancer	*N* = 138 878 responses from 155 hospital trusts across the National Health Service in England	Quantitative questionnaires: National Cancer Patient Experience Survey (NCPES)	• Survey included questions related to: Sex, employment status and ethnicity	The Index of Multiple Deprivation (IMD), the official composite measure of deprivation in England, was derived on the basis of patient postcode ascertained from the health record. This was done to ascertain whether the patient was categorised as deprived	Response rate of 63.9%
19.	Platonova	2016	USA	Community Setting	Integrated care: Patient-centered medical home (PCMH)	*N* = 548 patients	Cross sectional study Quantitative questionnaires: multi-item Consumer Assessment of Healthcare Providers and Systems scales developed by the Agency for Healthcare Research and Quality	• Patients were approached by research staff while waiting for their appointments • Multiple languages: The survey was available in English and Spanish. Survey • items were translated from English into Spanish by professional translators and reviewed by Spanish-speaking healthcare professionals • Some screening questions were removed or rephrased to shorten the survey, reduce the complexity and to reduce the reading level	Study conducted in 2 independent free clinics in a large metropolitan area in the Southeastern United States	Response rate 66%
20.	Redshaw	2018	UK	Hospital	Maternity care: Care associated with stillbirth	*N* = 473 participated in the survey	Quantitative survey	• An information sheet in 18 non-English languages gave information regarding a contact number for the team		Response rate 30%
21.	Ryan	2018	USA	Community setting	Mental health: Promotion of mental health (Mindfulness) among Latina immigrant women	*N* = 24 women	Quantitative: pre and post test survey	• Spanish version of the surveys used and demographic information was collected • Trained bilingual interviewers administered the surveys to participants in separate sessions before and after the intervention’s five sessions • Participants received a gift card in the amount of 20 dollars for each survey completed	None	None reported
22.	Schinkel	2016	Netherlands	Community Setting	Integrated care: Exploration of patients’ preferred and perceived participation and doctor–patient concordance in preferred doctor–patient relationship on patient satisfaction	*N* = 236 patients	Quantitative questionnaires: Pre and post test design After signing the informed consent form in the waiting room, participants were given preconsultation questionnaire. Following the consultation with the GP, they were given a post consultation questionnaire	• Recruitment in waiting rooms • Recruited both Dutch and bilingual Turkish-Dutch assistants for data collection • Multiple languages: questionnaires available in Dutch and Turkish • The patient questionnaires were pilot-tested twice among low-educated and low-literate Dutch and Turkish-Dutch people to ensure that all items were comprehensible to the targeted populations	none	none
23.	Schutt	2020	USA	Hospital	patient navigators and services for chronic illness	*N* = 157 patients before and *N* = 378 patients after	Quantitative questionnaires: Pre and post test design Health care satisfaction surveys both before and after the program design	• surveyed by phone both before and after the program design • Multiple languages: Questionnaires were translated into Spanish and Portuguese	none	none
24.	Sharif	2019	USA	Community care	Integrated care: healthcare experiences of Cambodian American refugees and immigrants	*N* = 308 patients	Quantitative questionnaires: questionnaires and medical records from two community clinics in Southern California	• A bilingual Khmer research assistant described the study to each patient, obtained informed consent from any interested patients and administered the baseline questionnaire • Data were collected on the respondents’ sociodemographic information, including gender current age, age at US entry, year of immigration to the US educational attainment, religious affiliation, employment status, participation in Food Stamps program, total annual household income and household size	patients recruited from two community clinics (one which has 11 different locations and the other with one location) in Long Beach, California	none
25.	Shin	2020	USA	Hospital	Patient experience with clinical pharmacist services	*N* = 99 Patients	Cross sectional study design Quantitative questionnaire: Oxford Patient Involvement and Experience Scale	• Multiple languages: survey offered in English and Spanish • Clinical pharmacists read out loud a script which described the survey purpose (i.e., to get feedback about and improve pharmacist services), directions, and privacy procedures	The patients completed surveys in a designated area in the clinic, but away from their clinical pharmacist, and surveys were inserted into a sealed box	none
26.	Soo	2013	Canada	Hospital	Radiation therapy	*N* = 128	Quantitative questionnaire: patient satisfaction survey	• Multiple languages: Chinese version of the questionnaire • The survey was pre-tested on volunteers and staff members for construct validity	No information	none
27.	Yelland	2015	Australia	Hospital	Maternity care—views and experiences of immigrant women of non-English speaking background (NESB) giving birth in Victoria, Australia	*N* = 4516	Quantitative questionnaire:	• Multiple languages: survey available in Arabic, Vietnamese, Cantonese, Mandarin, Somali, Turkish • Women were posted a questionnaire six months following the birth, together with a covering letter, and a reply paid envelope for returning the completed questionnaire	None	None

**Table 2:** Summary of included studies- Mixed methods *N* = 9

	**Author**	**Year**	**Country**	**Setting**	**Aspect measured in the Patient Reported Experience measure: topics and relevant questions**	**Sample and population**	**Description of qualitative and quantitative data collection (i.e. survey)**	**Specific strategies employed to improve participation of CALD population**	**How were recruitment sites identified and examples of places**	**Evidence of effectiveness of strategy to increase CALD patient participation**
1.	Bains	2021	Norway	Hospital	Maternity care	*N* = 401 international migrant women, ≤ 5 years length of residency in Norway (giving birth in urban Oslo) answered the questionnaire (87.6% response rate)	Cross-sectional study Mixed methods: Face to face interviews and a modified version of the Migrant Friendly Maternity Care Questionnaire (MFMCQ) The original questionnaire was adapted to the health system setting of Norway and modified after inputs from pilot testing	• Eligible women were recruited either on admission for delivery or at the postnatal ward • Interviews conducted face to face in the women’s own language of choice after birth, using an interpreter when needed • Written translations of the questionnaire were provided in nine languages: Arabic, Dari, English, French, Norwegian, Somali, Sorani, Tigrinya and Urdu • Training workshops for all the research personnel. The interviewers met regularly to discuss challenges and experiences • The user representatives (from non-governmental organisations and relevant migrant communities) gave feedback on readability, validity and cultural sensitivity of the questionnaire before data collection. After data collection, preliminary findings were presented, and interpretations were discussed with user representatives	Interviews were conducted in the postnatal ward	Response rate (87.6% response rate)
2.	Damery	2019	UK	Hospital	Mental health: Psychological difficulties (distress) in patients with end stage renal disease Specific questions included in the survey exploring the patient’s ethnicity	Survey sent to *N* = 3730 eligible patients Purposive sampling for the interviews: *N* = 46 Patients with end stage renal disease (ENDS) interviewed	Mixed methods: cross-sectional survey and semi -structured interviews	• Postal survey developed for the project and included some validated measures to assess aspects of distress and emotional adjustment • Questions included in the survey explored: socio-demographic and clinical information (age, gender, ethnicity, time since diagnosis) • Patients could involve a carer/ family member to complete the survey and this could be indicated on the survey	No specific information	Response rate 27/9%
3.	Dang	2017	USA	Hospital	Patients’ experience with the mobile phone intervention for heart failure	*N* = 42 patients	Randomised control trial and longitudinal measurement at 1 month and 3 months Mixed methods: Patient interviews and survey	• A 31item survey was developed for the study • Multiple languages offered for the interviews: Spanish and English • Questions in the survey explored: sociodemographic information like age, gender, ethnicity	Patients recruited from the hospital	None reported
4.	Hyatt	2018	Australia	Hospital	Patients experience with a communication intervention package (comprising consultation audio-recordings and question prompt lists) especially designed for CALD patients	*N* = 18 patients completed the interview and *N* = 17 completed the survey	Randomised control trial Mixed methods: Patient interviews and surveys	• Consent to participate in the study was obtained in the patient’s predominant language	Patients recruited from the hospital	None reported
5.	Kaltman	2016	USA	Primary care	Mental health: Latina immigrants experience with a mental health intervention	Convenience sample of Latina immigrants (*N* = 28) with depression and/or posttraumatic stress disorder (PTSD) for primary care clinics that serve the uninsured	Post-intervention data collection Mixed methods: survey and interviews	• Multiple languages: Interviews conducted in Spanish • Bilingual staff members conducted the interviews and the analysis • $20 and $30 gift cards	The intervention was conducted at a community primary care clinic in an area that serves low-income, uninsured patients, many of whom are Latino immigrants Recruitment was done via posted flyer, referral by clinic staff, and outreach screening in the waiting room	None reported
6.	Liu	2017	USA	Hospital	Maternity care: Birth experience of immigrant women with an intervention designed for prenatal care	*N* = 39 Spanish women	Post-intervention data collection Mixed methods: Interviews and surveys	• Bilingual staff members conducted the interviews • Demographic information collected in the survey	Patients recruited from the hospitals	None reported
7.	McBride	2017	Australia	Community care	Patients experience with an integrated healthcare service for asylum seekers and refugees	Purposive sampling (*N* = 18) participated in the interviews and (*N* = 159) completed the surveys	Patient experience Mixed methods: Interviews and survey	• Bicultural workers with experience in cross-cultural research were involved throughout each stage of the project, including methodology design, the development of survey tools and interview guides, recruitment, and data collection • Participant Information and Consent Forms were available in community languages • Multiple languages- interviews conducted in the patient’s preferred language	Interviews were conducted in a private room at Monash Health and were digitally recorded with permission from participants. Interviews were conducted in participants’ chosen language, and accredited interpreters were used Clients discharged attended a discharge information session. This meeting was used to administer a client feedback survey with consenting clients	None reported
8.	Mendoza	2018	USA	Community setting	Integrated care: Healthcare experience	*N* = 419 Latina women immigrants is associated with satisfaction with health care	Mixed: Qualitative interviews and surveys	• A structured face-to-face interview conducted in Spanish, either in the respondent's home or a community-based site, based on the participant's preference • Respondents received $20 cash for their participation • Details on study procedures provided in Spanish • Spanish language translation methods of measures like Social mobility measures and satisfaction with care (the medical mistrust and acculturation scales already had a Spanish versions)	Participants were recruited from various community sites in New York City, and by flyers posted in designated areas in the target communities (e.g., apartment buildings and community-based agencies and service facilities)	None
9.	Torres	2020	USA	Community setting	Health intervention to reduce unhealthy alcohol use in Latino immigrant men	*N* = 73 completed the survey and *N* = 20 completed the in-depth interview	Randomised control trial Mixed methods: in depth interviews and surveys (pre and post-test)	• Study participants received $30 for each survey and interview completed • Demographic information of the participants was collected in the survey • All surveys were interviewer administered by a promotor	Participants in study were recruited from a community-based organization serving Latino immigrants in Seattle, WA. The organization served as a day labor worker center, and therefore many Latino immigrant men came to the organization seeking employment each day	None reported

**Table 3:** Summary of included studies- Qualitative (*N* = 50)

	**Author**	**Year**	**Country**	**Setting**	**Aspect measured in the Patient Reported Experience measure**	**Sample and population**	**Description of qualitative data collection**	**Specific strategies employed to improve participation of CALD population**	**How were recruitment sites identified and examples of places**	**CALD Relevant topic/question areas**
1	Abuelmagd	2019	Norway	Community	Diabetes mellitus care	*N* = 18 Immigrant Kurdish patient	Focus group discussion	• Patient recruited through Kurdish networks and common places where Kurdish population frequently visit like mosques and cafes • Research team-member who led FGD had a Kurdish background • Focus group discussion held in meeting room convenient for participant to attend	Sites were places in Oslo that the general Kurdish population frequently visits (Kurdish mosques and cafes)	Interview guide was developed based on study aims and previous research on non-Western immigrants with T2DM
2	Ahrne	2019	Sweden	Community	Maternity care: specifically, Antenatal care	*N* = 16 mothers *N* = 13 fathers Somali immigrants	Focus group discussion	• Focus group discussion held in locations where Somali migrants are present • Somali-speaking research assistant, interpreters and facilitators • Recruitment took place through existing networks within the Somali diaspora, public preschools and Child Health Centres • Data collection conducted in Somali language and translated to Swedish • Refreshments were offered	Sites were identified because many Somali people migrated and lived in those locations. Also, a third site was identified by the mid-wives who had experience of working in that area	None
3	Alkhaled	2022	Norway	Hospital	General health care experience	*N* = 20 Newly immigrated, Arabic speaking patients, Purposive sampling	In-depth interviews	• Participant information sheet and Consent form in Arabic language • According to the participants’ wishes, all interviews were conducted in their homes • Interviews conducted in Arabic • One Research team member had an Arab background and spoke Arabic. Other 3 researchers had an immigrant research focus	Sites were five hospitals. There is no information on how they were selected	Interview guide addressed the themes of linguistic competencies during communication, cultural issues such as values and beliefs, experience of pain, the role of the patient’s family, meals, and their experience in dealing with the Norwegian health-care system
4	Bitar	2020	Sweden	Hospital	Maternity care: evaluation of a communication app	*N* = 10 Arabic immigrant women who had used the App at least two times	Telephone interviews	• Participants had the choice of in-person or telephone interviews • Interviews were conducted during pregnancy in Arabic by the first author who had an Arabic background • Written informed consent in Arabic was obtained	Sites were six antenatal clinics in southeast of Sweden where app was launched and used by participants	• Demography questions included: • Ethnicity • Years of residence in Sweden • Ability to communicate in Swedish
5	Carlsson	2016	Sweden	Hospital	Maternity care: Experiences and preferences of care following a prenatal diagnosis of congenital heart defect among Swedish immigrants	*N* = 9 Pregnant immigrants and their partners were consecutively recruited following a prenatal diagnosis of a congenital heart defect in the foetus	Interviews	• Participants were given the option to either be interviewed together with their partner, or individually. All couples chose joint interviews • The second author, a female sociolinguistic researcher with previous experience of conducting face-to-face interviews and with no clinical contact with the participants, conducted all five interviews in Swedish. Four Interviews conducted with the aid of a professional interpreter	Site was a tertiary referral centre for foetal cardiology. There is no information on how it was selected	Demography questions included: • Country of birth
6	Cervantes	2021	USA	Hospital	Hospital care: Experiences of Latinx individuals who were hospitalized with and survived COVID-19	Purposive sampling *N* = 60 Latinx adults	Semi structured telephone interviews	Interviews were conducted in English or Spanish according to participants’ preference	Identified participants via a data query that provided the contact information for individuals who self-identified as Latinx and had been hospitalized for COVID-19, and had an interviewer call them	Interview guide was developed based on a literature review of race disparities and the COVID-19 pandemic, with a particular focus on Latinx communities
7	Chu	2005	Australia	Hospital	Maternity care: Postnatal Care	*N* = 55 Participants. Three Chinese immigrant groups (People’s Republic of China (PRC), Hong Kong and Taiwan)	face-to face and telephone interviews of over 25 key informants in-depth face-to-face interviews (using an interviewing guide) with 30 women in their homes field visits to identified community organisations; and focus group discussions	• The project team was multi-disciplinary and multi-lingual in nature • The author employed three research assistants, one each from Taiwan, Hong Kong, and Mainland China • The informant had read and signed an informed consent form in the Chinese language	Participants were recruited through referral by community organisations and the researchers’ own social networks The informants were first approached by telephone, and upon receiving their expressed willingness to participate, were followed up with a home visit at a time nominated by them	Interview Guide questions included: • general background and migration history of informants • general health conditions before and after immigration; health beliefs and health utilisation behaviour • reproductive health beliefs, behaviour and experience in Australia
8	Decker	2021	USA, Mexico	Community, Hospitals	Maternity Care	*N* = 74 pregnant and/or parenting adolescents (Mexican origin)	Interviews, focus group discussion	• A binational team of trained and experienced researchers from Mexico and the United States conducted all focus groups and interviews in the language preferred (Spanish or English) • In recognition of their time and input, respondents received a $20 gift certificate in California while in Mexico, participants received infant supplies, such as diapers, per local institutional recommendations	• Guanajuato, Mexico, was identified as a traditional point of origin for migrants to California and Fresno, California, was identified as a primary point of arrival for Mexican immigrants • Youth in California were recruited from several community-based organizations serving pregnant and parenting youth • Study researchers used previously established relationships with clinics and organizations in both communities, and staff at these sites recruited youth to participate when they sought services at these sites	None
9	England	2003	United Kingdom	Primary Care	Paediatric Care	*N* = 24 mothers, Kurdish and Turkish refugees	Focus group discussion	• Consent form was provided in the appropriate language • Consent to record the sessions was obtained both in written form and verbally, as literacy levels for written Turkish are very variable among the mainly Kurdish patients • All groups were provided with crèche facilities and refreshments • ‘Health visitors’ and Turkish speaking ‘health advocates’ already working at the clinic. The focus groups were run by the health visitor and a health advocate, both of whom received focus group training prior to the study. In addition, another advocate acted as the interpreter	Site is a general practice surgery in North London where 18% of the attending patients are Turkish speaking Kurdish refugees	None
10	Falbe	2017	USA	Primary care	AHF (Active and Healthy Families) program to reduce obesity disparities in Latino children	*N* = 23 parents (Latino immigrants)	In – depth interviews	Interviews conducted in the participant’s preferred language (Spanish or English)	Parents participating in AHF in two clinics in San Francisco Bay area were chosen to participate	Interviews with AHF participants honed in specifically on “Promotora” qualities, actions, and relationship with patients (“Promotoras” in the trial were Spanish-speaking Latina mothers recruited to engage families, facilitate discussions and understanding of content)
11	Frahsa	2020	Switzerland	Community	Healthcare & social-services for chronic health conditions	Purposive, priori-defined maximum-variation sampling strategy **1. ***N* = 12 Turkish, *N* = 12Portuguese, *N* = 12 German Immigrant women with chronic health conditions **2. ***N* = 12 Swiss women with chronic health conditions	Multi-method qualitative Study: Semi-structured interviews, Focus Group Discussion Stakeholder Dialogues	• Interviews were conducted in Turkish, German and Portuguese by 4 bilingual researchers based at Swiss and Turkish universities • Interviews conducted at venues convenient to participants • Interviewees had option to be accompanied by members of their family or acquaintances • Participants received a gift upon completion of the interview • FGD was conducted in the language most comfortable for the greatest number of participants. In addition, a translator was present during the FGDs upon request by the participants • FGD participants received cash incentive and travel costs were covered	• Study took place at Swiss cantons of Bern and Geneva • Recruitment strategies to reach interviewees were: personal contacts via researchers’ professional and private networks, cultural associations, labor unions, associations for the elderly and retirement homes, academic institutes, hospitals, physiotherapists, and physicians or specialists known to have many immigrant patients and/or command of those patients’ native languages • Also recruited via public leaflets in shops, restaurants, pharmacies, churches etc., social media advertisements, and through snowballing by interviewees	• Any discrimination faced while living in Switzerland? • Length of stay in in Switzerland?
12	Garcia-Jimenez	2019	USA	Primary Care	Telephone Interpreter Services (TIS) at urban community clinic	Purposive Sampling *N* = 13 Spanish speaking immigrants who utilized TIS (*N* = 7 Ecuadorian, *N* = 2Columbian, *N* = 3 Dominican Republic)	Focus group discussion	• Focus group dates and times were varied in order to increase the number and diversity of participants • The focus groups were held in the clinic in order to minimize travel barriers to participation • The focus groups were facilitated in Spanish by the primary care resident physician on the research team (native speaker) • 2 research-team members were native speakers	• Site was an adult primary care clinic at the urban community clinic affiliated with the institution that approved and funded the project • Eligible individuals were recruited by means of flyers, identification by primary care providers, and face-to-face and telephone-based encounters	Focus Group Guide **Barriers & Facilitators:** 1.Using an interpreter telephone during a clinic visit makes me feel...... – Why? 2. Do you agree or disagree: Using an interpreter telephone is better than using my limited English. – Why? 3. What has been your experience with telephone interpreter use? 4. What makes it easy to use a telephone interpreter? 5. What makes it hard to use a telephone interpreter? 6. Do you like using the interpreter phone? – if YES, why – if NO, why 7. Do you agree or disagree: My doctor does a good job using the interpreter telephone. – How so? **Cultural Barriers:** 1. How does using the telephone interpreter affect your relationship with your doctor? 2. Does using a telephone interpreter make you feel differently about the care you receive? 3. Do you prefer your doctor speak Spanish, even if Spanish is their second language, to using the telephone interpreter? – if so, why – if not, why 4. Do you tell your doctor everything if you are using an interpreter phone? – if YES, why – if NO, why 5. What are your thoughts about the interpreter on the telephone? 6. How does the telephone interpreter services compare to other interpreter services? Using a clinic staff member? Using a family member? Baseline Questionnaire included: • Country of origin • English speaking ability • Year of immigration to USA • I have had providers who speak Spanish (Y/N) • My current provider uses an interpreter phone (Y/N) • If given the option, I would use a family-member instead of a telephone interpreter (Y/N) • In the past 12 months I have used an interpreter phone in _______ encounters
13	Garrett	2008	Australia	Hospital	Conceptions of cultural competency in acute health care	*N* = 49 patients from non-English speaking backgrounds (NESB) *N* = 10 carers from NESB [Arabic, Italian Vietnamese, Mandarin, Cantonese]	Focus group discussion	• Participant candidates were invited by bilingual research officers to attend the language-appropriate focus group • PI and a bilingual research officer co-facilitated each FG, which was conducted in the relevant community language, with a professional healthcare interpreter formally interpreting proceeding	The study was undertaken at a tertiary hospital serving a large multicultural community in the western suburbs of Sydney	Focus groups topics included: • impact of limited English proficiency on hospital experience • access to interpreters
14	Gilbert	2019	Australia	Community	Australian healthcare system & transnational treatment options	Purposive Sampling *N* = 34 Indian Immigrants	Focus group discussion	• FGD were conducted in various community spaces • They were usually run after-hours or on weekends, to best avoid conflicting with participants' work schedules • FG were facilitated by bilingual Hindi researcher • Participants each received A$20 compensation for their time, light refreshments provided • FG with older group was conducted in Hindi	• Melbourne was chosen as the city of interest as it has the largest Indian population of any Australian city • Participants were recruited through advertisements placed in community spaces such as local libraries and community centres • Various community groups ran presentations introducing the study	This study focuses on recent Indian migrants to Australia; their negotiation of the Australian healthcare system and their use of transnational treatment options. By exploring this through a framework of trust, researchers demonstrate how socio-cultural discrepancies in the ways trust is reached in the Indian and Australia healthcare systems respectively results in deficits of trust between Indian migrants and Australian healthcare professionals
15	Gronseth	2006	Norway	Community and primary care	Health and sickness as embedded in social life and cultural values	Interviews *N* = 100 Tamil refugees (70 families and 30 single individuals) Observations 50 health consultations	In-depth interviews, participant observations including healthcare consultations	• The researcher was a consultant in the Psychosocial Team for Refugees in Northern Norway • In the early stages of the research project, the researcher worked with Tamil interpreters, Tamil refugees who had obtained reasonable skills in Norwegian • When researcher was left alone with informants, they often volunteered supplementary, which tended to include additional emotional and bodily expressed information • When conducting the second stage of research based on the full year of fieldwork, she made less use of interpreters in order to avoid the kind of formality caused by the situation. A methodological approach of ‘being with’ and sharing experiences, or ‘attending’ to the field, was emphasized. a Broader spectrum of data sources was used, including verbal and cognitive data based on interviews and observations, but also the informants’ (and her own) bodily expressions, sensations and perceptions She took part in daily activities like cooking, eating, celebrations and ceremonies, watching Tamil TV and interacting with children. Within this approach, the researcher relied on Norwegian as the language of communication. Some Tamils spoke the language fluently, others rather poorly, and some produced close to no Norwegian. For those who spoke poorly, family members would help out in conversations, and in some instances the author arranged for an interview in which she called upon an interpreter	The site is a small fishing village in along the arctic coast of Norway with a Tamil refugee resettlement	Bi medicine vs Holistic medicine (Ayurveda)
16	Hadwiger	2005	USA	Community	How acculturation is manifested in illness narratives of diabetes	Mexican/Mexican -American adults No information on numbers	Ethnography involving formal interviews, participant observation and case-studies	• A network sample was recruited through previous contacts in Hispanic community • The interview guide was translated into Spanish • Two bicultural research -assistants participated as interpreters during interviews • Most interviews were conducted in informant’s home • Significant others were allowed to be present at the preference of the informant • Informed consent was obtained through a Spanish consent form	• This particular county in Midwest USA recorded a significant rise in Spanish-speaking population within 2 years following the establishment of a new agricultural plant • Primary field work included an internship with county health department’s bilingual bicultural health educator, participation in Hispanic religious affiliations, involvement with community Latino center, informal interviews with health providers in the community, & identification of community stake-holders	Contextual Data: • Duration lived in USA • Generational Status • Language proficiency • Acculturation Ratings: Cuellar, Arnold and Maldonado’s 2nd Acculturation Rating Scale for Mexican Americans (ARSMA-2)
17	Herrero-Arias	2021	Norway	Community	Norwegian healthcare system	*N* = 20 Southern European immigrant parents	In-depth interviews and Focus group discussions	• Interview locations were chosen by participants (home/office) • FGD were moderated by a Spanish researcher	• Data was collected from 3 Norwegian municipalities with both rural and urban areas with high concentration of immigrants • Participants recruited through Facebook, RHA’s personal network, attendance of gatherings organized by immigrant communities in Norway and snowball sampling	Demography questions included: • Country of origin • Years lived in Norway
18	Hogg	2015	United Kingdom	Primary care	Health-visiting service for families with young children	Purposive sampling *N* = 16 Pakistani immigrant mothers *N* = 15 Chinese immigrant mothers	Semi-structured interviews	• Bilingual research assistants assisted in recruitment • Interviews were conducted at participants’ homes • Interviews conducted by bilingual research assistants, or with the help of interpreters	No information provided	• Experience of health visiting service including cultural sensitivity • Language • Generational Status • Experience of living with extended family
19	Jager	2019	Netherlands	Primary care	Dietetic care in Type 2 diabetes	*N* = 12 diabetic adults who are immigrants from Turkey, Morocco, Iraq and Caraco; and visit a dietician	In-depth semi-structured interviews	• Recruitment done by dieticians and researchers in practices orally and by information leaflets in different languages • Interviews held in preferred language thorough interpreters who were specially trained • Interviews conducted in participants’ home • Family present in several cases	Recruitment was done from nine Dutch dietetic practices in areas with a high proportion of migrant residents	• Country of origin • Length of stay in Netherlands • Explanatory model of illness by Arthur Kleinman was one of the models on which topic list was based. It addresses the social and cultural influences on illness and health
20	Janevic	2020	USA	Hospital	Impact of perceived racial-ethnic discrimination on patient-provider communication	*N* = 16 Latina women who gave birth in a large hospital and had attended prenatal care at the same hospital’s clinic	Focus group discussion	• Participants were offered a $100 gift card for their participation • All materials were translated into Spanish • Focus group was conducted in Spanish language • Moderators were of a similar racial—ethnic background as study participants and were trained in the content of the discussion guide	A large hospital in New York. There is no information on why this site was selected	• Patient-provider communication during childbirth among Latina women was investigated from the perspective of Critical Race Theory (CRT). CRT focuses on the social construction of race and recognizes the pervasiveness of structural racism • The research team developed a discussion guide containing a series of questions on the women’s experiences during their birth hospitalization, communication with providers, and **if they perceived differential treatment for any reason** • Examples of questions include: “Was there a doctor, nurse, or other health care provider during your time in the hospital with whom you felt uncomfortable asking questions? Tell me more about this experience.”; “Can you describe any time during your care you may have felt you were treated differently from other women? Why do you think you we**re treated differently?”**
21	Jomeen	2013	United Kingdom	Community	Maternity care	*N* = 219 women who self-identified as coming from black, ethnic minority (BME)groups in a national survey of women who had given birth in the last 3 months	Telephone interviews	• A leaflet in a wide range of languages with a Freephone number was mailed • Women could choose to participate by telephone interview or with the help of a Languageline interpreter	Data collected by the Office for National Statistics using birth registration records	No information provided
22	Jonkers	2011	Netherlands	Hospital	Ethnicity-related factors contributing to sub-standard maternity care and its effects on severe maternal morbidity among immigrant women	*N* = 40 immigrant women	In-depth interviews	• Most interviews conducted at participants’ homes 2-6 weeks after discharge, in hospital for those with prolonged hospitalisation • Interpreters were offered but accepted by a single participant only • Husbands, partners, relatives and friends involved during the complication participated in almost all interviews and added their perspectives. Sometimes they also acted as interpreters	Recruitment was done from a bigger country-wide registration study	None
23	Jun	2018	USA	Community	Pre-natal genetic testing and decision-making process	Referrals and Snowball sampling *N* = 10 Korean-American women	Face-to face or phone interviews	• Research team had three bilingual, bicultural scholars • Two bilingual research-team members translated interview-guide from English to Korean and conducted interviews in Korean language • Interviews were conducted either face-to-face or by phone, depending on where participants lived • Gif-cards were provided	• Participants initially recruited by referrals from Korean community leaders in a Midwestern urban area • Subsequently, a snowball sampling technique was used to recruit additional participants • Due to snowball sampling, participants were dispersed geographically throughout US	• No. of years living in USA
24	Kumar	2018	United Kingdom	Hospital	Satisfaction towards receiving information about biologics and future support preferences for Rheumatoid arthritis (RA)	Purposive sampling *N* = 27 South Asian patients with RA	Semi-structured interviews: Face-to-face or phone	Interviews conducted in preferred language (Punjabi/Hindi/English) by multilingual researcher	Sites were secondary care rheumatology clinics at 2 large hospitals	None
25	Kwok	2017	Australia	Community	Influence of cultural values and language on Treatment-decision making (TDM) process in Breast cancer	*N* = 23 Chinese-Australian women	Focus group discussions	• FGD conducted in native languages (Mandarin or Chinese) by first author and a trained research assistant, both registered nurses with clinical oncology experience and fluent in English, Mandarin and Cantonese • FGD conducted in premises where cancer support groups met	Participants were recruited from a cancer support group serving the Chinese community	• Confucian philosophy was used as a conceptual framework. It powerfully influences and behaviour of Chinese people -central concepts include deep respect for authority figures and mutual dependence within families along with responsibility for individual family members • English proficiency • Years in Australia
26	LaManusco	2016	USA	Community, Primary Care	Maternity care (Perinatal Care)	*N* = 14 Karen (Burmese) refugee women	In-depth interviews	• All documents were in Karen language • Interviews conducted at home, alone or in pairs according to participant preferences • Interviews were conducted in Karen with help of interpreter • Participants received $20 for participating	• Buffalo, New York, where a community of 4000 Karen refugees continues to grow • Primary care site was community health centre for Karen refugees	• Social contextual model (SCM): there are multiple psychosocial, population, and structural/environmental factors that influence health behaviours (Interview guide based on this and lit/rv) • Interviews with Karen perinatal patients focused on experiences during pregnancy, labour, and the postpartum period in Burma, Thailand, and/or Buffalo; women’s questions about and opinions of perinatal care in Buffalo; challenges faced during the perinatal period; and Karen perinatal traditions
27	Madden	2017	United Kingdom	Community	Healthcare experiences to understand barriers to engagement	Purposive Sampling, Snowball Sampling *N* = 42 East European immigrants (¾ Polish)	In-depth interviews (individual and small group), Focus-group discussion	• Adverts in Polish & English • All recruitment materials (posters and leaflets), the participant information sheets and consent forms were translated into Polish by a native speaker • Gatekeepers and participants were asked if they would like these translated into other languages when interviews and focus groups were arranged, however, this was not requested • Face-to-face interviews were conducted at place of participants’ preference (including cafes and community centres) • To encourage open and easy conversation, in only one language, focus groups were conducted with participants who already knew each other • Participants assisted each other with translation during Focus group discussion	• A medium town in North England • Recruitment done through local Polish and East European networks, general services like social housing providers, local authority services, recruitment agencies, a drug and alcohol service and libraries	• Nationality
28	Maneze	2016	Australia	Community	Communication issues in interaction with healthcare practitioner (HCP)	Purposive & Snowball sampling *N* = 58 Filipino immigrant adults with at least 1 chronic disease	Focus group discussions	Focus-group discussions moderated by bilingual researcher	• Recruitment was carried out in the Greater Western Sydney area where the Filipino migrant population was known to be the largest • Leaders and members of Filipino community organisations were invited to participate • Participants were asked to provide contact details of the researchers to friends and family members who they felt might be interested	• Questions focused on the facilitators and barriers to health-seeking and communication issues experienced during clinical encounters
29	Markovic	2004	Australia	Hospital	Gynaecological cancer disclosure and treatment decision-making	*N* = 11 immigrant women (Europe, Asia & Pacific, Middle-East) with gynaecological cancer	In-depth interviews, Participant Observation in cancer support group and out-patients’ dept	• Interpreters were offered for interviews where required but women preferred family interpreter for reasons of confidentiality • Richness of women’s responses indicated their general readiness to talk openly with the assistance of a family interpreter. The use of a family interpreter provided with the opportunity to explore gate-keeping with regard to cancer disclosure practice • An interview conducted with a professional interpreter, known to the woman through community networks, again demonstrated that she was not inhibited by the presence of ‘an outsider’	• A tertiary, public hospital in Melbourne, Australia. There is no information on how this site was selected • Rapport established with female inpatients by visiting them on the ward and attending outpatients’ clinic when they presented for follow-up appointments, participating in a hospital-based cancer support group	• Continent of origin
30	McKinlay	2015	New Zealand	Primary care	Multimorbidity (MM)healthcare in a Very Low-Cost Access (VLCA) general practice	*N* = 5 patients of Cambodian origin, and *N* = 5 patients of Samoan origin	Interviews and focus-group discussions	• A Pacific Navigator (a role designed to enable Pacific patients and family to access health services to improve health and wellbeing) individually approached and recruited Samoan patients, and similarly, a Cambodian interpreter approached and recruited Cambodian patients • Trained interpreters each facilitated language-specific focus groups (Cambodian & Samoan) • Individual patient interviews were undertaken in the patients’ homes and focus groups at a church near the general practice which is regularly used for health promotion activities	• VLCC general practice in Wellington which manages a complex population with high levels of deprivation and a diverse ethnic mix	None
31	McLeish	2005	United Kingdom	Community	Maternity care	Convenience & snowball sampling *N* = 33 refugee women	Semi-structured Interviews	Women were interviewed either at home or in a neutral location such as a refugee/asylum support group, with an interpreter where necessary	• No information provided • Recruitment done through refugee/asylum support groups, refugee agencies, asylum accommodation providers and health professionals	None
32	Moxey	2016	United Kingdom	Community	Antenatal and intrapartum care in females exposed to genital mutilation	Purposive, Convenience and snowball sampling *N* = 10 Somali women who had accessed antenatal care services within past 5 years	Semi-structured interviews	• Interviews were conducted in a private room, offering women privacy to discuss a potentially sensitive topic • A lay female interpreter, identified and trusted by the community, was present where required • All participants received an inconvenience allowance in the form of a £10 shopping voucher at the end of the interview	• Participants recruited from 2 community centres in Birmingham, England • This location was identified due to the large resident Somali community and high numbers of Somali women with FGM accessing ANC services locally	• No. of years in England
33	Muscat	2018	Australia	Hospital	Healthcare decision- making in Renal care	Stratified, purposive sampling to represent dominant cultural & language groups *N* = 35 adults of CALD background receiving in-centre haemodialysis for advanced chronic kidney disease	Semi-structured interviews	Arabic interviews were facilitated by bilingual facilitator who was research assistant trained in qualitative methods	Four large haemodialysis units in Greater Western Sydney, Australia where 38% of population is born overseas	Interview Guide Experience of decision-making (renal replacement therapy and other); information and decision-making preferences, and; **cultural values**
34	Nadeau	2017	Canada	Primary care	Youth Mental Health Services in a collaborative care model	*N* = 5 migrant young patients (12–17 years old) who received mental health services in a collaborative setting for at least 1 year *N* = 5 parents of migrant young patients	F2F Semi-structured interviews	• Interviews with youths or parents were conducted either at the family’s home or at the local service centres (called CLSCs), according to their preference, • One parent interview was conducted in presence of an interpreter as requested • Youth and family members were offered compensation for their time (a $10 gift certificate or $20, resp)	Study site was primary-care, community based. health and social service center in Montreal that serves a multiethnic population and has three CLSCs.. These centres offer proximity services in multiple locations (such as the CLSCs themselves, schools, or patients’ homes)	• Ethnicity
35	Nguyen	2022	USA	Hospital	Outpatient telemedicine	Purposive sampling *N* = 15 patients speaking Cantonese or Spanish [*N* = 6 were Asian/Pacific Islander/other, *N* = 11 were Hispanic/Latino]	Semi-structured telephone interviews	• In order to obtain a range of experiences, English, Spanish, and Cantonese-speaking patients who were scheduled for telemedicine visits were purposively sampled • Participants were interviewed by bilingual study staff in their preferred language • Demography data was collected by trained research-assistants, 2 of whom were bilingual • Participants were reimbursed for their time up to $40	Three clinics—general medicine, obstetrics, and pulmonary–within the San Francisco Health Network, which is the public healthcare delivery system in the city and county of San Francisco, California, serving almost exclusively Medicaid and other uninsured/publicly-insured patients	• Preferred language • Self-identified race and ethnicity
36	Northridge	2017	USA	Community	Dental Care at 2 dental school clinics	*N* = 69 Dominican immigrants (50 years and older) *N* = 53 Puerto Rican immigrants (50 years and older)	Focus group discussion	• Both field recruiters were bilingual in English and Spanish and had several years of experience working with racial/ethnic minority older adults and senior center directors in upper Manhattan • Group discussions were held two locations where participants did not need to travel far from their residential neighbourhoods • All participants were offered the services of a taxi driver to pick them up at their homes or at a senior center, bring them to the focus group, and take them home afterwards. This strategy was crucial for ensuring focus group attendance, particularly for older adults with mobility problems • Groups were conducted in Spanish by senior qualitative researcher who was fluent in Spanish, along with bilingual assistant moderator	• Senior centres in upper Manhattan were chosen rather than places where older adults receive dental care in order to obtain a sample of individuals who did not necessarily have access to, or seek, dental care • Senior centres have been identified as important “third places” (as distinct from homes or “first places” and worksites or “second places”) where older adults may be targeted for health promotion activities	• Race/ethnicity • Primary language
37	Peters	2019	Netherlands	Community	Maternity Care	Purposive sampling, Snowball sampling *N* = 86 immigrant women	Interviews	• The locations of the focus group sessions were chosen by the participants • Assistance was available for respondents with a limited Dutch language proficiency. If needed, field experts and one of the moderators were available to interpret (languages: Papiamento, Turkish, Moroccan Berber, Portuguese and Moroccan Arabic) and further explain questions asked by the moderator	• Rotterdam is the second-largest city of the Netherlands with relatively high proportion of low educated inhabitants, with relatively high levels of unemployment, income segregation and poverty compared to other large Dutch cities • Active recruitment methods, including by ‘verbal advertising’ and through social networks. (a) peer education meetings organised by the community health workers, (b) primary schools during coffee breaks, (c) secondary schools and a community college during health(care) educational lessons(d) neighbourhood community centres	• Ethnicity • Years living in the Netherlands • Language proficiency level • Generational level
38	Ranji	2012	Sweden	Hospital	Ultrasound examination in second trimester of pregnancy	9 Farsi speaking couples (*N* = 18)	Semi-structured individual interviews	• Seven interviews took place at the parent’s home and the other two interviews were held in a room at the university • All of the parents were interviewed in the Farsi language by the first author who is a native Farsi-speaking midwife • Each woman and her partner/husband were interviewed separately in order to give them freedom to speak their true feelings in confidentiality	A University hospital with southernmost region of Sweden as its catchment area. There is no information on how the site was identified	None
39	Rose	2015	Australia	Community	Self-management support from GPs	*N* = 28 ethnically diverse diabetes patients attending group diabetes education	Group interviews	A bilingual health worker who was knowledgeable in diabetes, acting as an interpreter for the Arabic-speaking and Vietnamese-speaking groups, was present during the interviews	Three community education groups for people with type 2 diabetes located in a culturally diverse region in Sydney	None
40	Semedo	2020	Sweden	Primary care	Multimodal pain rehabilitation (MMR) programme	*N* = 7 Somali women	Semi-structured interviews, focus group discussion	• Invitation letter was written in Swedish and Somali • All individual interviews and FGD were conducted in the meeting room at the healthcare centre where the MMR programme took place • Somali interpreter was available	The site was a healthcare centre in Northern Sweden where a group of Somalian women underwent an MMR programme	None
41	Shaw	2016	Australia	Hospital	Cancer care coordination	*N* = 18 immigrant patients/carers [*N* = 8 Chinese, *N* = 5 Arabic *N* = 5 Macedonian]	Telephone interviews, Focus group discussion	• A letter of invitation together with information about the study in the patient’s own language and in English was mailed to all eligible patients • Patients were also invited to have their caregiver accompany them to the focus groups • Bi-lingual researchers telephoned potential participants who indicated an interest in the study after the mail-out to provide further study information • Participants were given the option of either a focus group or an interview • Written consent was obtained in patient’s own language • Researchers fluent in Cantonese, Mandarin, Arabic or Macedonian conducted interviews & FGDs • Bi-lingual researchers were experienced in health research, group facilitation and/or local community support group facilitation. They received training for the study and were supported by more experienced qualitative researchers who attended the focus groups and reviewed the interviews • Travel and associated costs of participation were covered • FGDs were conducted at a community library close to the hospitals, as this location was convenient for participants	Sites were 2 Cancer centres at 2 metropolitan hospitals in Sydney. There is no information on how these sites were identified	• Country of birth • Years in Australia
42	Singh	2020	Canada	Community	Compassion in healthcare	Convenience & theoretical sampling *N* = 19 South Asian immigrants	Semi-structured interviews	Interviews were conducted in either English, Hindi or Punjabi according to participants’ preference by a multilingual research-team member	No information provided	Demography Questions: • Fluency in English – If no, specify language fluent in – Hindi or Punjabi • Immigration status: • Canadian born • Immigrant: (< 5 years, 5–9 years,10–19 years, ≥ 20 years) • Temporary resident • Spirituality/Religiousness • Spiritual and religious • Spiritual but not religious • Religious but not spiritual • Neither • Religion Interview questions targeted both general aspects of compassion and cultural and ethnic perspectives regarding the concept, experience, importance and the facilitators and barriers of compassion 1. Considering your cultural background, what does compassion mean to you personally? 2. Can you tell me a little bit about one of your recent healthcare experiences where you felt you either received care that was compassionate or lacked compassion? 3. Can you describe an example of when you experienced care that you felt was compassionate? 4. What were the key parts that made it compassionate? 5. Can you give me an example of when you experienced care that you felt lack of compassion? 6. What were the key parts that made it uncompassionate? 7. How do you think, for example, your understanding of compassion might differ from a person from a different cultural background? 8. What do you feel are similarities across cultures? 9. What advice would you give your health care providers on providing compassion to members of your cultural community? 10. How can we make our healthcare systems more compassionate, especially towards the members of your cultural and religious community? 11. Is there anything that we have not talked about day that we have missed or you were hoping to talk about?
43	Speed	2021	United Kingdom	Community	Primary care	*N* = 28Chinese migrants 60 years and over with cardiovascular disease or significant risk factors (hypertension/high cholesterol)	Focus group discussion	• FGDs were held in preferred language (Mandarin/Cantonese/English) • FGDs which were held in a private room in a local community centre	Local community of a “Chinatown” in the Northwest of England	• Place of birth • First language
44	Valibhoy	2017	Australia	Community	Mental health services	Purposive sampling *N* = 16 young (18–25 yrs.) refugees	Interviews	• Because this small population is ‘‘hard to reach,’’ researchers expected recruitment would be a gradual process, requiring a multifaceted approach • A multistage informed consent process was used, allowing time for consideration before scheduling an interview e. Anecdotally, the barriers that refugee youth face to mental health service utilisation, especially stigma and language, also appeared to pose barriers to research participation • Three participants were recruited and interviewed with prebriefed, qualified interpreters	Participants were recruited from ethno-specific/cultural diversity services, nongovernment specialist services, public and community mental health services, a government support service, an education support service, an Internet site, volunteers, word-of-mouth, and snowballing	• Country of birth • Years lived in Australia • No. of languages known • Religion
45	White	2019	Australia	Hospital	Delivery of culturally competent healthcare in acute hospital setting	*N* = 12 immigrants [Greek, Chinese Dari, Vietnamese] who were admitted and received services from a trained interpreter on at least one occasion during one hospital admission had another admission without one	In-depth interviews	• Patients meeting inclusion criteria were mailed a letter of invite to the study, including study information, in their preferred language • After 2 weeks the researchers, using relevant interpreters, phoned each patient to answer questions and schedule interview • Interviews were held at a convenient time and location, typically the patient’s home	General Medicine program at Monash Medical Centre (MMC) or Dandenong Hospital (DH)	• Languages known
46	Wieslander	2015	USA, Mexico	Hospital	Factors affecting disease understanding among women with pelvic organ prolapse (POP)	*N* = 36 immigrant women with symptomatic POP	Focus group discussion	• 4/8 FGDs were held in Spanish with the help of bilingual moderator • A small honorarium was offered to patients for their time	Speciality practices at 3 large medical centres	• country of origin • religion
47	Wilkinson	2017	United Kingdom	Hospital	End-of-life care in renal disease	*N* = 16 South Asian renal patients	Interviews	• Majority of the members of research-team were bilingual in the main South Asian languages spoken in the UK, and conducted the patient interviews • Interviews were conducted in the participant’s preferred spoken language and at their choice of location, which was usually their home	Four UK centres providing kidney care to diverse populations: West London, Luton, Leicester and Bradford	end of life care for South Asian patients across haemodialysis, peritoneal dialysis (PD) and conservative care pathways, to identify where there are inequalities in access and experience of end-of-life care
48	Wojnar	2015	USA	Community	Maternity Care (Perinatal Care)	*N* = 48 Somalian immigrant men and women who arrived in US within the past 5 yrs., had a child or children born in their homelands or refugee camps and at least one child born in the United States	Semi-structured individual interviews, Interviews with couples, Follow-up phone interview	• Couples expressed interest in the study by calling a Somali interpreter hired to assist with the investigation. The interpreter explained the study purpose and procedures, answered questions, obtained a verbal consent to meet for an interview, and determined which families would require interpretation services to conduct the interviews • Interpreter planned an in-person interview • between the investigator and the prospective participants for an in-person interview • Interviews were conducted in preferred language Somali language or Bantu (a dialect spoken by Somali Bantus) • All interviews were conducted at locations convenient for the study participants. The majority of interviews were conducted in the participants’ homes. Four interviews were conducted at a community center that allowed for complete privacy	• All of the participants resided in the Pacific Northwest • Several recruitment strategies were used: personal solicitation through a community partner agency that offers educational and social programs to new immigrants, provider referrals, and snow-balling technique	• Interview questions included: • Tell me about your life after you settled in the United States? • Pregnancy What are some special customs observed by Somalis in pregnancy? What was is like for you as expectant parents in the USA? How was the prenatal care different from your homeland/the refugee camp? How was the prenatal care similar in your homeland/refugee camp? • Childbirth What are some special customs observed by Somalis during childbirth? How was childbirth process different from your homeland/refugee camp experience? How was it similar to your experience in your homeland/refugee camp? • Postpartum What are some special customs observed by Somalis in postpartum period?
49	Wurth	2018	Switzerland	Hospital	difficulties patients with migration background and healthcare professionals experience in their shared clinical encounters and to explore ethical aspects involved	*N* = 16 Albanian immigrants *N* = 16 Albanian immigrants	Ethnography: participant observation (patient encounters with medical staff) and semi-structured interviews of patients	Language during interviews was based on the same conditions as during medical consultations: Most interviews were conducted in German, if patients had used an interpreter (professional or non-professional) during medical consultations, the same interpreter was also involved for the interviews	Sites were two outpatient clinics at large hospitals There is no information on how these sites were identified	• The semi-structured interviews combined a set of pre-defined questions developed from the literature study and questions that were triggered by the clinical experience of two practicing physicians in the research team in cross-cultural encounters • Interview questions were supplemented with observations made during the clinical encounter under examination • Each interview covered sections on demographics, cultural and social aspects, language and communication
50	Zehetmair	2021	Germany	Primary care	Mental healthcare at a psychosocial walk-in clinic	*N* = 22 refugees [*N* = 5 Eastern Europe *N* = 12 Asia *N* = 5Africa]	Semi-structured interviews	Recruitment and Interviews were conducted in preferred language with use of interpreter	A medical and psychosocial walk-in clinic run by Heidelberg University Hospital at Heidelberg, Germany. It supports psychologically burdened refugees within the state reception & registration center “Patrick Henry Village” (accommodating around 1200 newly arrived refugees and asylum-seekers)	• country of origin • religion

**Table 4:** Summary of included grey articles (*n* = 11)

	**Author/ organisation**	**Publication type**	**Year**	**Country**	**Key points**	**Evaluative data**	**Link**
1.	Australian Commission for Safety and Quality in Health Care	Review (“Patient experience and satisfaction surveys conducted within public and private hospitals in Australia”)	2012	Australia	• Available in multiple languages and formats (using pictures/symbols) • In the Northern Territory hospital survey, meaningful pictures and symbols were incorporated within surveys • In Victoria, the surveys were available in English and 16 community languages	None	https://www.safetyandquality.gov.au/sites/default/files/migrated/Review-of-Hospital-Patient-Experience-Surveys-conducted-by-Australian-Hospitals-30-March-2012-FINAL.pdf
2.	New South Wales Health Agency for Clinical Innovation	Information sheet (“Patient and staff experience – Patient Experience Trackers”)	n.d	Australia	• Online method of data collection • The experience tracker is a fast and effective way to collect patient feedback • Patients who are cognitively impaired or not able to answer the questions may have their identified carer complete the survey	None	https://aci.health.nsw.gov.au/__data/assets/pdf_file/0005/235265/F-CLD-PETS.pdf
3.	New South Wales Health Agency for Clinical Innovation	Online resources (“Patient Reported Measures: resources for clinicians and patients”)	2017	Australia	• Available in multiple languages • Fact sheet on providing feedback via the patient reported measures was available in different languages	None	https://aci.health.nsw.gov.au/statewide-programs/prms/resources
4.	Clinical Excellence Queensland	Statewide report (2019) (“Your Experience of Service”)	2020	Australia	• Multiple languages and multiple formats of delivery • The YES survey is available for completion in paper form in 24 languages, and on tablet devices	YES Experience report (2019): *n* = 20,429 service episodes 3,474 surveys were completed and returned for analysis	https://clinicalexcellence.qld.gov.au/priority-areas/patient-experience/your-experience-service-yes
5.	Health Quality & Safety Commission	Report (“Measuring culturally safe care through the patient experience surveys”)	2021	New Zealand	• A special set of cultural safety questions were designed for the CALD population that were included in the patient experience survey	None	https://www.hqsc.govt.nz/assets/Our-data/Publications-resources/Measuring_culturally_safe_care_through_peX_PDF_April_2021.pdf
6.	Health Quality & Safety Commission	Report (“Development of patient experience indicators for New Zealand”)	2013	New Zealand	• Multiple methods of collecting patient experience • Patient surveys can be distributed in multiple languages	None	https://www.hqsc.govt.nz/assets/Our-data/Publications-resources/KPMG-patient-experience-indicators-Aug-2013.pdf
7.	Ministry of Health	Report (“Patient Experience (2011/12): Key findings of the New Zealand Health Survey”)	2011	New Zealand	Survey collected demographic information and Interviews are conducted using computer-assisted personal interviews (CAPI) (see page 51–51). Sampling strategy to allow surveys to be completed by culturally diverse ethnic groups	*n* = 12,596 adults completed the survey *n* = 4,558 children’s primary caregivers completed the survey	https://www.health.govt.nz/system/files/documents/publications/patient-experience-2011-12-key-findings-of-new-zealand-health-survey-sept13-v3.pdf
8.	National Health Service (including NHS England, NHS Scotland and NHS Wales)	Website (“Friends and Family Test (FFT)”)	2020	United Kingdom	• Multiple languages offered for the patient experience survey (NHS England» Making the Friends and Family Test inclusive) • Video with easy-to-follow animations to encourage patient participation in the patient experience surveys • An information video using sign language	None	https://www.nhs.uk/using-the-nhs/about-the-nhs/friends-and-family-test-fft/
9.	National Health Service (including NHS England, NHS Scotland and NHS Wales)	Website (“National Patient and Staff Surveys”)	n.d	United Kingdom	• All surveys had a question asking about the patient race/ethnicity. (GP Patient Survey – Faq (gp-patient.co.uk)) No other specific detail enquired	None	https://www.england.nhs.uk/statistics/statistical-work-areas/patient-surveys/
10.	The Kings Fund	Report (“Ethnic Diversity and Mental Health in London: recent developments”)	2003	United Kingdom	• Clearly outlining the need to collect culturally relevant data • No specific questions outlined	None	https://www.kingsfund.org.uk/sites/default/files/field/field_publication_file/ethnic-diversity-mental-health-london-recent-developments-frank-keating-david-robertson-nutan-kotecha-kings-fund-1-august-2003.pdf
11.	The Beryl Institute	Video (“Equity, Bias and Human Experience”)	n.d	USA	• Staff training video specifically designed to improve patient experience in relation to equity and bias	None	https://www.theberylinstitute.org/page/LearningBites

### Characteristics of included studies

#### Database search

Studies in the peer-reviewed literature (*n* = 86) originated from: the United States of America (US; *n* = 29), Australia (*n* = 16), the United Kingdom (UK; *n* = 13), Sweden (*n* = 6), Norway (*n*  = 5), The Netherlands (*n* = 4), Canada (*n* = 4), Germany (*n* = 3), Switzerland (*n* = 2), New Zealand (*n* = 1), Denmark (*n* = 1), Greece (*n* = 1), multiple European Union (EU) countries (*n* = 1). Studies were conducted in: hospitals (*n* = 38), community settings (*n* = 29), primary care (*n* = 14) and dental services (*n* = 2), or in more than one setting (*n* = 3). Patients’ experiences were explored in relation to: maternity (*n* = 21), integrated care and/or care coordination (*n* = 12), mental health (*n* = 7), general practice (GP) (*n* = 3), emergency care (*n* = 2), gynaecology (*n* = 2),, pharmacy services (*n* = 2), convalescence care (*n* = 1), end of life care (*n* = 2), dental care (*n* = 2), student directed clinic care (*n* = 1), paediatric care (*n* = 1), radiation therapy (*n* = 1) and hospital care for COVID-19 (*n* = 1).

Of the total 86 studies identified from the electronic database searches, 27 studies used quantitative methods via survey, 50 employed qualitative methods and nine employed mixed methods combining surveys and interviews. Of the 50 qualitative studies, 37 (74%) used a single method of qualitative data-collection: individual interviews (*n* = 26) [14–39], focus-group discussions (FGD) (*n* = 10) [40–49] or group interview (*n* = 1) [50]. Thirteen of the 50 qualitative studies used two or more methods for data collection [51–63] combining individual interviews with FGDs (*n* = 9) [52, 53, 55–59, 63], with participant observation (*n* = 3) [54, 60, 62], with group interviews (*n* = 1) [61] or with FGDs and a site visit (*n* = 1) [40]. Almost all studies were with adults aged 18 and older, except for two studies that included samples aged 12–20 years old. Experiences were reported directly from patients (*n* = 85), or including both patients and carers/support persons (*n* = 8) aside from one study collecting end of life care experiences from the next of kin [64]. Of the studies that employed survey methods, 12 were cross sectional [65–76] and eight were experimental; four pre- and post-intervention studies [77–80], three randomised control trials (RCTs) [80–82], and one longitudinal study (1 and 3-months post intervention) [81]. Sample size ranged from 24 to 138,878 in the quantitative studies and 9 to 219 in the qualitative studies. Seven population-based studies had samples of > 1000 participants and included a subset of people from ethnically diverse backgrounds, identified via demographic survey items.

#### Grey literature

The grey literature search yielded 11 documents that originated from Australia (*n* = 4), New Zealand (*n* = 3), the UK (*n* = 3) and the US (*n* = 1). Of the four documents originating from Australia, one was aimed at a national level, developed by the Australian Commission on Safety and Quality in Health Care [83] and three were aimed at state level, developed by the NSW Agency for Clinical Innovation and Clinical Excellence Queensland [84–86]. The two documents from New Zealand were aimed at the national level, both developed by the Health Quality and Safety Commission New Zealand [87, 88]. In the UK, the identified documents were from the National Health Service (NHS; which includes NHS England, Scotland and Wales) [89, 90] and The Kings Fund [91]. The document from US was developed by The Beryl Institute [92]. Five of the grey literature documents were reports on patient experience surveys, of which three explored services and care in mental health settings [84, 89, 91] and two explored health care experiences system-wide [89, 93]. Three documents explored patient experience and satisfaction items, indicators, and survey instruments used for patient experience measurement [83, 87, 88]. Two resources were fact sheets about the importance of collecting patient experiences and instructions about how to complete them [85], and one was a video discussing the importance of the collecting diverse patient experiences [92]. One report described a survey instrument which was used to collect real-time data via a tracker device with specific discussion of responding to diversity via the support of carers, although ethnically diverse populations were not explicitly identified [86].

### Review findings

Evidence from the 97 included papers was explored in relation to the review aim to identify the approaches and techniques employed in PREMs to improve participation amongst people from ethnically diverse backgrounds. Findings led to the development of four key themes: i) strategies for identifying and recruiting ethnically diverse communities to participate in PREMs, ii) approaches to creating data collection instruments and processes to support PREMs completion by people from ethnically diverse backgrounds, iii) the patient experience topics of relevance to ethnically diverse communities, and iv) the application of patient experience data from ethnically diverse communities in healthcare. Findings are presented in relation to each of these four areas.

- i. Strategies for identifying and recruiting ethnically diverse communities

Gathering patient-reported experiences of people from ethnically diverse backgrounds is contingent upon identifying ethnically diverse communities and engaging these communities in PREMs. Evidence from the peer-reviewed literature indicated that patient experience data were often captured from individuals or families whilst in a clinical setting. In 49 studies, PREMs were captured while individuals were visiting, being admitted to, or discharged from, hospitals [14–17, 21, 23, 25, 29, 31, 33, 36, 37, 44, 49, 51, 52, 54, 60, 62, 63, 65, 68, 73, 76, 81, 82, 94–96] or while individuals were visiting primary care clinics [18–20, 26, 30, 38, 42, 43, 54, 58, 59, 69, 70, 75, 78, 97–102]. Thirty-one further studies reported PREMs being gathered via community and/or online environments. For example, ethnically diverse respondents were often identified through community organizations or via attending social events using flyers and social media [72, 80, 98, 99, 102, 103]. Other methods for identifying ethnically diverse communities included using administrative data. Given the limited sociocultural data available regarding ethnically diverse communities, these methods were based on identifying ethnically diverse communities from lower socio-economic groups such as via the Index of Multiple Deprivation (IMD) in England to identify eligible people based on their postcode and derived diversity [104], or via birth registration records from the Office of National Statistics. Identified individuals were then mailed leaflets in multiple languages to seek ethnically diverse respondents [22]. One study sought experiences of refugees aged 18–25 years using mental health services, which used multiple modalities for recruitment (e.g., project flyers on noticeboards, mail-outs, and presentations to professionals and youth from refugee backgrounds).a multi-faceted approach [35].

Qualitative studies often used additional sampling techniques to try to access relevant ethnically diverse participants, which were often directed to a particular community or group of communities who had access to a specific service or were experiencing a specific health condition. Twelve studies used purposive sampling to reach their target population [14, 19, 25, 28, 29, 31, 32, 35, 45, 47, 53, 57], including stratified, purposive sampling to represent the dominant cultural and language groups in their community. Snowball sampling was reported in five studies [24, 27, 32, 47, 57] and convenience sampling was reported in two studies [27, 28]. Community leaders were used to identify potential respondents in two studies via referrals [24, 47].

Bicultural workers were often engaged in the process of identifying and recruiting ethnically diverse communities. Twenty-nine studies reported the involvement of multicultural and/or multilingual team members or community leaders. Bicultural workers included researchers, multicultural health workers, community networks, formal and informal interpreters. Bicultural workers contributed to projects at a range of stages. In the establishment of projects to aid identification and recruitment of ethnically diverse participants. Seven studies discussed the involvement of multicultural and bi/multilingual staff members in facilitating recruitment [19, 33, 44, 48, 58, 61, 63]. The strategies identified involved contacting eligible ethnically diverse participants via telephone or speaking to potential participants in person to explain the study purpose and to obtain informed consent. Materials were frequently created in ethnically diverse community languages required to aid recruitment of communities, with bicultural workers also supporting the recruitment process. Seventeen studies developed documents in community languages and/or engaged bilingual personnel in the consent and recruitment process to support language translation of study information [14, 15, 19, 20, 33, 36, 42, 44, 48, 51, 53, 55, 57–59, 61, 63]. Ten studies provided patient-information sheets and consent documents in relevant community languages [14, 15, 20, 36, 42, 51, 55, 57, 59, 63]. Bicultural staff also aided in developing interview and focus group discussion guides [19, 33, 44, 48, 58, 61, 63]. In three studies, bicultural staff were trained in preparation to contribute to study processes; and three studies discussed provision of training to bilingual research personnel [42, 46, 63]. The training was in relation to support the personnel’s involvement in data-collection [42, 46] and one study trained staff for recruitment and data collection [63]. Six studies reported providing cash incentives to patients [69, 70, 77, 80, 98, 103]. The grey literature provided little detail about how patients from ethnically diverse backgrounds might be identified or recruited to complete PREMs.

- ii. Creating data collection instruments and processes to support completion

Several strategies were used to support the process of data collection of patient experience. Twenty-five of the 36 studies included surveys that had been translated into multiple languages to aid completion [65, 67–79, 81, 94, 97–100, 102, 103, 105–107]. Whilst using translated surveys was considered to enable a wider population to complete the surveys, no direct evidence of the impact of using translated surveys on the quality and quantity of data collected was reported.

Just over half of the surveys used (20/36; 56%) were previously validated, English-language instrument(s) [64, 65, 67, 71, 72, 74–77, 79, 94, 98, 101, 103–106, 108, 109]. Of which, seven were adapted based on pilot testing with a subset of the target population [65, 67, 72, 76, 94, 106, 109]. Changes that resulted from pilot testing of existing instruments were: modifications to the response options to respond to diversity [72, 109], increasing the appropriateness of the tool content for ethnically diverse communities [76, 106], and reducing the complexity and reading level [75]. Three studies reported using shorter versions of a survey to increase completion rates [75, 94, 97], with response rates ranging from 38 to 60% in these studies.

A further 10 studies created purpose-built PREMs for ethnically diverse populations [66, 68–70, 73, 78, 80, 81, 96, 100]. Of these, five PREMs were developed drawing items together from a range of existing validated surveys [66, 69, 70, 80, 100], and the remainder were developed as new surveys [69, 70, 96]. Six of the studies that developed new surveys reported pilot testing the surveys with the target population to ensure appropriateness and comprehension [68–70, 73, 78, 96]. Three studies reported conducting reliability or validity analyses [68, 75, 76].

Five documents from the grey literature discussed adaptations to PREMs data collection instruments to aid completion by diverse respondents. Two documents provided insight about PREMs administration with a variety of existing mainstream PREMs in Australia i.e. Victoria Patient Satisfaction Monitor, Picker survey, National Healthcare Agreement, Queensland patient survey, Press Ganey and Hospital Consumer Assessment Healthcare Providers and Systems (HCAPS). These documents proposed the use of a range of data collection methods via health services, mail, online, and telephone interviewing to respond to diverse population needs [83, 84]. Similar approaches to diverse data collection modes were identified by the Health Quality & Safety Commission New Zealand [87]. The Health Quality & Safety Commission New Zealand created additional questions toconsider the assessment of culturally safe care through patient experience measurement [88]. A comprehensive approach was undertaken to develop culturally safe and appropriate questions, which involved exploring available frameworks, designing questions and cognitive pre-testing of questions to check for relevance, importance, and clarity with a diverse group of patients. The resulting additional patient survey questions captured experiences of the following aspects of care: listening, engaging in shared decision making, kindness and comfort, respect, and recognition of personal needs (cultural, spiritual and individual). Other grey literature documents identified additional modalities for PREMs, including a patient experience tracker; a computer-based tool to collect real-time patient experience data [86], the use of computer-assisted personal interviews (CAPI) [93], and offering PREMs in multiple languages [84, 87, 89].

In addition to creating or adapting survey instruments, flexibility in the process of data collection was a feature of studies aiming to capture PREMs from ethnically diverse communities. In survey studies, PREMs were administered via face-to-face, verbal surveys (*N* = 10) [67, 69–71, 74, 78–80, 94, 101, 105], via telephone verbal surveys (*N* = 2) [96, 109], paper-based surveys (*N* = 9) [64–66, 74, 82, 100, 105, 107, 109], and online (*N* = 2) [70, 74]. The mode of data collection and location appeared to be associated with the completion rates in these studies. Response rates were reported in 16 survey studies, which ranged from 20 to 95%. Generally, surveys with higher response rates (> 70%) were completed on-site in hospitals/clinic waiting areas and/or completed as verbal surveys conducted face-to-face. The survey with the highest response rate of 95% included only three items [99]. The lowest response rates (< 30%) were for paper-based, postal surveys. [66, 109, 110]. Interview studies adapted data collection methods to increase uptake among ethnically diverse communities by conducting interviews in an individual’s preferred language (*n* = 7) [65, 80, 81, 95, 96, 98, 103] and in a location convenient for them, often going into communities (*n* = 4) [65, 95, 96, 103]. Studies also offered participants the choice of completing [15, 16, 26, 63] individual interviews or focus groups [63], or of interviews being completed face-to-face or via telephone [15]. Participants were also offered the option to conduct individual interviews or interviews in pairs [26].

Bicultural workers were often used to support the data collection process in addition to identifying and recruiting ethnically diverse populations. Bicultural workers supported data collection (*n* = 24) [14, 15, 20, 21, 24, 25, 31, 33, 34, 37, 42–44, 46–48, 50–53, 56, 58, 61, 63] and data analysis (*n* = 12) [14, 15, 24, 31, 33, 43, 44, 47, 52, 53, 55, 56]. Most (86%; 43/50) of the qualitative studies conducted data collection in the respondents’ preferred community language via bicultural workers or interpreters [14, 15, 17–22, 24–35, 37–39, 41–50, 53–56, 58–63]. In addition to supporting language and cultural needs, the need for flexible and supportive data collection processes to enable ethnically diverse communities to engage in PREMs was reported in several studies. Strategies included scheduling focus groups at a range of times, including evenings or weekends [43, 45], providing transport for participants to and from the data collection site [48], and providing childcare facilities [42]. The opportunity to include a support person/s was also identified as helpful in the data collection process and comprehension of materials. Seven studies reported enabling a suppport person, such as a family member, to be present during interviews or focus groups [16, 20, 23, 53, 55, 59, 63], and four survey studies invited the involvement of a support person to assist respondents to complete PREMs [66, 67, 102, 109].

Support for the process of collecting PREMs among participants from diverse communities, in the grey literature, was outlined in four resources. One organisation provided fact sheets translated into multiple language for patients about how to share their experiences [85]. While another resource was a video with easy-to-follow animations that encouraged patients from ethnically diverse backgrounds to participate in PREMs [89]. The collection of patient experiences through an underpinning lens of equity and bias was identified in two resources, which included a brief video for staff members to address and promote an understanding patient experience through the lens of equity and bias [88, 92].

- iii. Patient experience topics of relevance for ethnically diverse communities

The included studies showed that most PREMs captured standard healthcare experiences commonly featured in mainstream population surveys, however aspects specific to ethnically diverse populations were notable. These were: healthcare navigation and access [64, 67–70, 72, 75, 78, 96, 97, 99–103, 105, 106, 109], care experiences of models and processes of care designed to be culturally relevant [65, 73, 74, 77, 79, 80, 94, 95, 98, 107, 110], cultural competence of healthcare teams and services [74, 78, 96, 97], experiences of patient-provider communication [64, 69, 70, 72–76, 82, 94, 100, 104, 106, 107, 109, 110], and of respect in the context of patient and clinician interactions [73, 74, 80, 101, 109, 110].

Health system navigation and access are topics of central focus in studies exploring migrant experiences of healthcare, but evidence of the impact of health system navigation and access problems experienced by migrants were apparent within studies of a range of services. Key examples are shown in relation to reliance on emergency departments for the treatment and management of chronic conditions and cancer due to lack of knowledge and understanding about how to access the health services via primary and outpatient care [68, 70]. Poorer experiences of models and processes of care that have been established for the mainstream population were also notable among migrant populations, with evidence of a lack of shared understanding of patient education materials, processes to coordinate care, and the actions expected of patients in their own care in service areas such as maternity and cancer care [63, 65, 67]. Poorer experiences of care were often reflected in low scores on patient satisfaction measures [67, 68].

Topics of importance further differed based on key features of ethnically diverse communities, such as ethnicity, country of origin and English language proficiency. For example, communities in which English language proficiency was low identified challenges in health system navigation and communication with healthcare teams more so than for groups with good English language proficiency [82]. In communities with low English proficiency, a language concordant health professional was identified as increasing satisfaction with care experiences [41, 97]. Communities that were well established within the host country also indicated fewer problems in understanding and accessing the healthcare system [82]. Cultural competence in relation to specific healthcare issues, such as the experience of cancer and of maternity care, in which expectations may vary based on cultural and religious background, emerged as important to consider in evaluating experiences of care [16, 23, 41, 51, 82]. Low healthcare engagement of migrant communities and opportunities to develop skills that enable self-management were also identified as factors contributing to poor healthcare experiences in these areas of care [23, 41].

Care coordination emerged as a critical topic for ethnically diverse communities, but was connected with the quality and availability of interpreter services and access to suitable health service information [63, 69]. Topics of importance to ethnically diverse communities may therefore include experiences of care coordination, particularly in relation to chronic conditions, maternity care and cancer, but require consideration of the features of additional support that are needed to create a positive experience. For example, experiences may be impacted by whether interpreters available for *all* aspects of care in which they were required, easy to access by the patient and of a good quality such that the patient and professional generated a shared understanding of the situation and next steps.

Communication and respect in the patient-professional dyad was a notable topic for ethnically diverse communities in their experience of care. Evidence from studies of chronic disease management demonstrated that the quality and clarity of communication with healthcare professionals (or lack of) was a key contributor to their healthcare experiences [53]. Studies that evaluated culturally tailored interventions, such as a family-based obesity intervention, further demonstrated that when people had culturally appropriate communication they had more positive experiences of care [18]. A series of language-specific focus groups revealed key indicators of a positive patient experience for ethnically diverse communities were compassionate, kind and respectful treatment, and the negotiated involvement of their family in health care decision making [44]. Lack of culturally competent behaviour among health professionals was perceived as leaving patients feeling powerless in their own care [44].

- iv. Applications of PREMs from ethnically diverse communities in healthcare

Patients’ experiences were gathered and reported predominantly to understand experiences of services of new programs and interventions targeted to ethnically diverse communities (69%; 59/86 studies). Five of these studies gathered PREMs seeking to improve capability in intercultural communication among healthcare staff [65, 73, 105], in person-centred care and shared decision making that is responsive to patient diversity [73, 104] and for managing patient distress [66]. In these studies, PREMs data was used to create educational materials and programs for staff training and development. Beyond these five studies, the evidence generated from the reviewed literature was not described in terms of its application. While not being directly applied to create change, 33 studies did provide recommendations for future health system planning to improve experiences among the ethnically diverse community based on their PREMs results [14, 16, 17, 19, 24, 25, 28, 30, 34–39, 41, 42, 44, 46, 47, 49, 51–53, 57, 58, 61, 65, 67, 72, 80, 82, 104, 105]. Areas for action included improving patient-professional communication, understanding of culturally respectful behaviours [16, 23, 51–53], access to interpreters [16] and personalised and culturally relevant patient education [20, 23, 37, 40, 44, 46, 51, 53, 63]. The grey literature predominantly provided PREMs to be used for evaluating service delivery rather than reporting applications of PREMs with ethnically diverse communities to change care [83, 84, 89, 93].

**Fig. 2:**
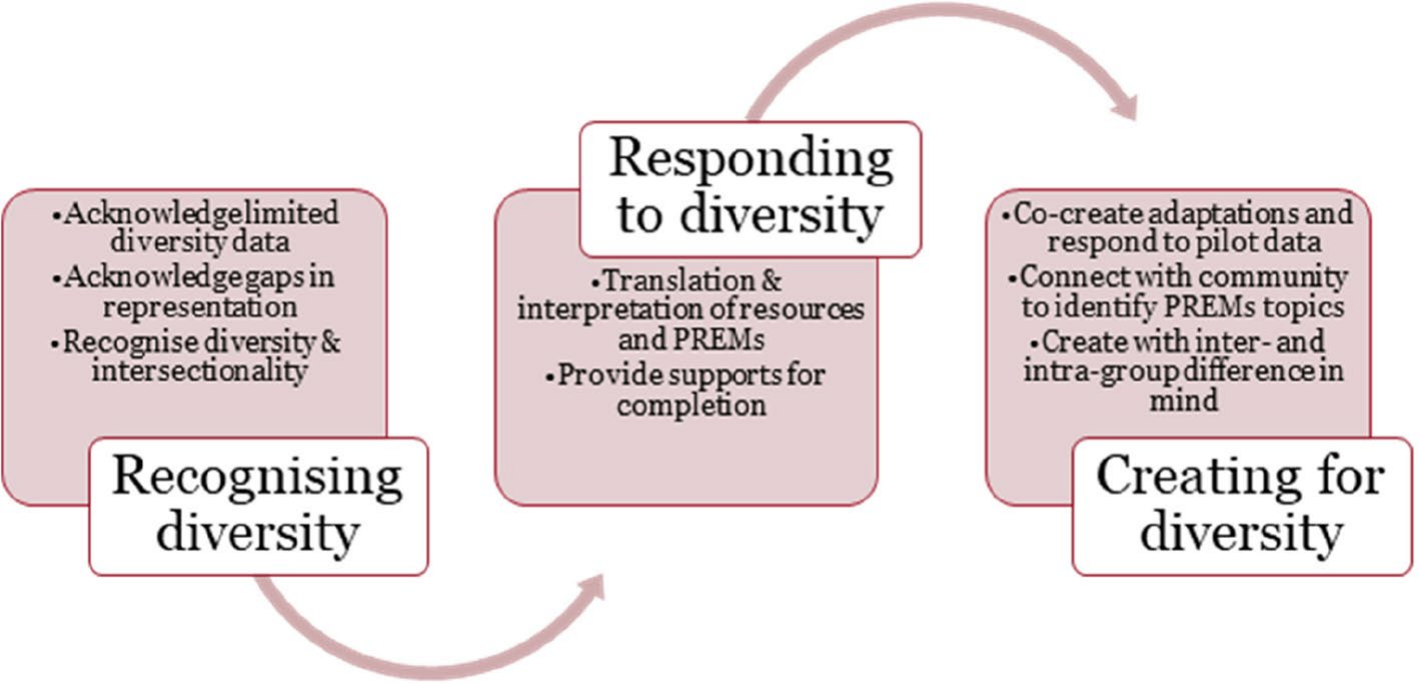
Maturity model for diversity in PREMs

Based on the review findings, a preliminary maturity model (Fig. 2) was produced to guide short- medium- and long-term action, which may have application to other diverse population groups.

## Discussion

A substantial literature demonstrated the methods and approaches used to collect patient-reported experiences among ethnically diverse communities, with 97 papers included in the review. Data extraction and synthesis revealed five broad strategies employed throughout the process of PREMs development and collection that sought to increase uptake and completion of PREMs with a range of communities in community and healthcare settings. These five strategies were: 1) ensuring the project design was responsive to culturally and linguistically diverse needs when establishing the project team and processes, 2) targeting resources and communication about the purpose, process and application of PREMs, 3) embedding flexibility into processes of collecting PREMs, 4) creating inclusive data collection processes relative to the community needs and preferences, and 5) recognising the direct and indirect costs with reimbursement and remuneration.

Central to most identified strategies, was collaboration with bicultural/multicultural staff and community members who provided critical support in one or more stages of PREM design and collection. Bi/multi-cultural staff and/or community members contributed to creating and communicating information about the purpose of PREMs, supporting translation to ensure high quality, advising on suitability of the content and its complexity with reference to community needs and pilot testing materials. Engaging bi/multicultural team and community members to elicit patient-reported experiences offers a number of benefits, but requires consideration and planning at the outset of PREMs work to ensure mutual learning, empowerment, and adequate resourcing [111, 112]. Cross-cultural research highlights a range of considerations when working over cross-cultural boundaries [112].

As health systems move towards value-based care, PREMs are increasingly important as one of the key indicators to demonstrate that health services are improving what matters to patients [113]. Almost half of the Australian population have ethnically diverse backgrounds with one or more parents born overseas, making PREMs participation from these communities essential to provide representative data [114]. Recent evidence indicates that ethnicity is one factor that may be assocaited with different patient experiences [115]. This review demonstrates that there are methods that are commonly and successfully used to increase responses from ethnic minority groups. When considering the strategies that have been used to greatest effect, it is apparent that additional resource is required at all stages of PREMs collection to increase community engagement and responses. Requirements for public health services to achieve a representative sample of respondents are lacking along with the tools and resources to enable this. Despite strong traction towards value-based care, without these implementation supports, communities will continue to lack appropriate PREMs tools and the support needed to engage in their completion.

### Implications

In the context of existing PREM instruments and processes, the maturity model resulting from this review has several implications for progressing PREMs engagement among ethnically diverse populations. Firstly, the model highlights the need to be able to recognise diversity in population groups. Limited and incomplete socio-cultural data is a barrier to this in many health systems worldwide [2]. The Australian context provides an example of how the need to capture information on diversity has advanced at a system level. In 2022, in addition their minimum core set of data variables (country of birth; main language other than English spoken at home; proficiency in spoken English, and Indigenous status), the Australian Bureau of Statistics (ABS) recommended collection of: country of birth of father and mother, religious affiliation, and year of arrival in Australia to increase the ease of identification of key features of diverse communities and factors that may impact on health and social well-being. Ensuring comprehensive routine capture of socio-cultural data via standardised data variables is a central system requirement to support better understanding of and provision for societal needs [107]. Access to these data may help to identify and facilitate comparison of PREMs results between services, cohorts and over time to track improvement where comparable measures are used.

With the target population identified, the second stage of the model identifies the need to respond to diversity via flexible and supported data collection methods. A wide literature exists on inclusive and cross-cultural survey content and methods, which demonstrates key aspects of survey behaviour that may be targeted to increase access and minimise bias in uptake, completion and outcomes from PREMs with ethnically diverse communities [108, 109]. Finally, the model depicts the need for a shift in the conceptualisation and design of PREMs that ensures measurement tools and processes are created to include diversity, with collaborative and community-partnership approaches championed. Aligning with the premise of person-centric healthcare, it is proposed that by developing PREMs topics, instruments and methods with community members, resulting tools and processes are likely to better suit community needs and preferences, leading to greater participation.

### Limitations

As an REA, the search scope was limited to three electronic databases and relevant material may have been omitted from the review. Although a wide range of search terms were developed in consultation with a medical librarian who is experienced in constituting and executing literature searches, the concept of experience is broad and relevant material, which may have also constrained the sensitivity of the search process. The inclusion of grey literature enabled this review to identify current approaches used in health systems for PREM collection and the extent to which these have sought to respond to diversity.

## Conclusions

A large literature has reported experiences of healthcare among ethnically diverse populations that demonstrate approaches that can be used to increase participation in PREMs collection among diverse communities. Five strategies have been predominantly used to increase PREMs participation, most often reported in research studies. Strategies include being responsive to community needs, targeting recruitment resources and communication, embedding flexibility in data collection processes, creating inclusive data collection processes, recognising the costs of participation and providing remuneration. Whilst strategies are available that appear to be effective in encouraging minority ethnic groups to participate in PREMs, there was limited evidence of their application in surveying conducted by public health system organisations. Public health systems must dedicate resource to enable the engagement of bi/multicultural health staff and community leaders to design and support the administration of PREMs if they are to ensure representative participation in PREMs across communities. In the longer-term, by working in collaboration with their communities, systems and services may co-create targeted PREMs that enable uptake and completion among communities.

## Data Availability

Data sharing is not applicable to this article.

## Abbreviations

ABS: Australian bureau of statistics
CALD: Culturally and linguistically diverse
FGD: Focus group discussion
OECD: Organisation for economic co-operation and development
PREM: Patient reported experience measure
USA: United States of America
UK: United Kingdom
